# Headaches and lymphocytic pleocytosis after COVID-19 require the exclusion of encephalitis

**DOI:** 10.1186/s42466-026-00461-z

**Published:** 2026-02-03

**Authors:** Josef Finsterer

**Affiliations:** Neurology Department, Neurology & Neurophysiology Center, Postfach 20, Vienna, 1180 Austria

Letter to the Editor

We read with interest the article by Umathum et al. about a 75-year-old woman who presented with headaches, dizziness, and symmetrical proximal tetraparesis [1]. The examination revealed absent Achilles tendon reflexes, decreased patellar and arm reflexes, axonal neuropathy, mild pleocytosis, and an intraatrial thrombus [1]. The patient’s medical history revealed a SARS-CoV-2 infection 7 weeks earlier [1]. Methylprednisolone was ineffective, and the patient died, presumably of a myocardial infarction, 12 days after admission [1]. The study is interesting but worthy of discussion.

First of all, we disagree with the assumption that the patient did not have central nervous system (CNS) involvement during her lifetime [1]. The patient complained of holocrine headaches and dizziness, had pleocytosis, and the autopsy revealed lymphocytic infiltration [1]. These findings support the assumption that the patient did indeed have cerebral involvement. Most likely, she suffered from either infectious or immunological encephalitis. Mild pleocytosis is a strong argument for viral encephalitis. Therefore, we should know whether the cerebrospinal fluid (CSF) was tested not only for SARS-CoV-2 but also for all other neurotropic viruses, whether the antibodies associated with autoimmune encephalitis were measured in serum and CSF and were negative, whether cytokines, chemokines, and glial cell factors were elevated in the CSF, and whether an intra-vitam MRI with contrast agent was performed and was truly normal.

The second point is that Guillain-Barré syndrome (GBS) of the Bickerstaff encephalitis subtype or GBS associated with viral or immunological encephalitis was not sufficiently ruled out. GBS is a common complication of SARS-CoV-2 infection [2]. Were the F-wave responses normal, or were the number, latency, and amplitude reduced? We should also know whether the CSF protein was elevated or not. Suspicion of GBS requires not only the exclusion of SARS-CoV-2, but also of numerous other viral and bacterial infectious agents, vaccinations, trauma, or previous surgeries [3]. Has the patients received virostatics or immunoglobulins?

The third point is that cerebral lymphoma was not sufficiently ruled out. Was there any evidence of malignancy in the lymphocytes detected in the basal ganglia and brain stem? Lymphoma can easily be overlooked [4]. Was there painless lymph node swelling, were the lymph nodes examined at autopsy, and were lymphoma cells detected? Did the patient complain of weight loss, severe night sweats, or chills?

The fourth point is that comorbidities and concomitant medications were not specified [1]. In the case of a 75-year-old woman, it is conceivable that her medical history already included a number of diseases. Knowledge of concomitant medications and comorbidities is crucial in order to assess whether these could have contributed to the fatal course of the disease.

The fifth point is that the course of the disease is not sufficiently described [1]. Did the SARS-CoV-2 infection manifest itself through symptoms or signs in the central nervous system (CNS) or peripheral nervous system (PNS)? Did she recover completely or incompletely from the SARS-CoV-2 infection? Since when has she suffered from headaches and dizziness, how many days after admission were CFS tests, nerve conduction studies, and echocardiography performed? Was the myocardial infarction due to an atrio-coronary embolism, was the stroke cardioembolic, and did she develop atrial fibrillation?

The sixth point is that there is no mention of how the intra-atrial thrombus was treated [1]. Was the patient anticoagulated or heparinized? Was the thrombus still found at autopsy?

In summary, encephalitis must definitely be ruled out in a patient with headache and pleocytosis who has recently had SARS-CoV-2 infection. Similarly, GBS should have been ruled out in a patient with tetraparesis who had recently had COVID-19 infection.

## Data Availability

All data are available from the corresponding author.
